# A Case Study of the South Puget Intertribal Planning Agency’s Comprehensive Cancer Control Planning and Community Mobilization Process

**Published:** 2008-03-15

**Authors:** Carrie Nass, John Simmons, Deborah Bowen, Teresa Guthrie

**Affiliations:** National Cancer Institute’s Cancer Information Service–Northwest Region, Fred Hutchinson Cancer Research Center; South Puget Intertribal Planning Agency, Shelton, Washington; University of Washington Fred Hutchinson Cancer Research Center, Seattle, Washington; National Cancer Institute’s Cancer Information Service–Northwest Region, Seattle, Washington

## Abstract

**Background:**

The high rates of cancer among American Indians and Alaska Natives are of growing concern.

**Context:**

In response to high cancer rates, national, state, and tribal organizations have worked to assess knowledge, attitudes, beliefs, and screening practices related to cancer in American Indian and Alaska Native communities and to increase awareness and use of cancer screening. The National Comprehensive Cancer Control Program (NCCCP) of the Centers for Disease Control and Prevention is one such effort. NCCCP's comprehensive cancer control (CCC) planning process provides a new approach to planning and implementing cancer control programs. The CCC process and components for American Indians and Alaska Natives are not yet fully understood because this is a fairly new approach for these communities. Therefore, the purpose of our case study was to describe the CCC process and its outcomes and successes as applied to these communities and to identify key components and lessons learned from the South Puget Intertribal Planning Agency's (SPIPA's) CCC planning and community mobilization process.

**Methods:**

We used interviews, document reviews, and observations to collect data on SPIPA's CCC planning and community mobilization process.

**Consequences:**

We identified the key components of SPIPA's CCC as funding and hiring key staff, partnering with outside organizations, developing a project management plan and a core planning team, creating community cancer orientations, conducting community cancer surveys, developing a community advisory committee, ongoing training and engaging of the community advisory committee, and supporting the leadership of the communities involved.

**Interpretation:**

The CCC planning process is a practicable model, even for groups with little experience or few resources. The principles identified in this case study can be applied to the cancer control planning process for other tribes.

## Background

American Indian and Alaska Native communities have significantly worse cancer rates and poorer access to cancer control interventions than do non-Native populations (1). Opportunities exist to reduce these disparities through special initiatives, such as the Centers for Disease Control and Prevention's National Comprehensive Cancer Control Program (NCCCP). The NCCCP's comprehensive cancer control (CCC) model is a collaborative process through which a community and its partners pool resources to promote cancer prevention, improve cancer detection, and increase access to health services to reduce the burden of cancer. The goal of CCC is to help reduce cancer risk, detect cancers earlier, improve treatments, and enhance survivorship and quality of life for people with cancer (2). A successful CCC planning process helps states and tribes to focus on cancer and to implement cancer control interventions; however, there is not yet a full understanding of how to conduct CCC planning successfully in tribal communities or of what planning components are.

## Context

The specific aims of our case study, conducted in 2005 and 2006, were to describe the CCC process, outcomes, and successes and to identify key components and lessons learned from the South Puget Intertribal Planning Agency's (SPIPA's) CCC planning and community mobilization process. SPIPA, created in 1976, is a five-tribe consortium headquartered in Shelton, Washington, that serves the Chehalis, Nisqually, Skokomish, Squaxin Island, and Shoalwater Bay tribes. SPIPA supports each tribe's vision of success and wellness by delivering social and health services through training, technical assistance, resource development, and planning (3). SPIPA is governed by its Board of Directors comprising tribal council members and representatives from each of the five SPIPA tribes.

American Indians and Alaska Natives in Washington State have a lower incidence of most cancers than does the total population (4,5); however, they have lower screening rates for cancer, more risk factors, and lower survival rates than the general U.S. population (4). There are many reasons for these disparities. Like many other American Indian and Alaska Native communities, SPIPA tribe members had a fear of the word *cancer* and often did not talk about it. Having the time and resources to plan for cancer control gave the SPIPA tribal communities time to talk about cancer, think about cancer, fight cancer, and support those with cancer. The Table describes the specific cultural considerations in the CCC planning process and how SPIPA responded to them.

## Methods

Our case study included direct observation of SPIPA's CCC planning process; in-depth semistructured interviews with 13 key informants selected from tribal health clinic staff, SPIPA CCC program staff, members of the SPIPA Community Advisory Committee, and tribal leaders not involved in the CCC planning process; and detailed review of meeting minutes. We used purposive sampling to identify the key informants for interview. Eleven (85%) of the 13 key informants selected were American Indian.

We recorded interviews with key informants either on tape or in writing. We analyzed these interviews in two ways, according to methods suggested by Yin for case study analyses (6). First, we grouped the interview responses by key informant type (e.g., tribal leader, community advisory committee member). We then counted the number of times similar types of answers came up on each question. We added the number of similar responses to give a rough estimate of the relative importance of the various components of SPIPA's CCC planning process that the key informants identified. To ensure reliability, wherever possible, we compared key informant interview data with observational data from the informants' involvement with the SPIPA CCC planning process. Key informants' rights were protected throughout the entire process. SPIPA does not have an institutional review board, so we received approval from SPIPA's Board of Directors and the University of Washington's Institutional Review Board.

## Consequences

Analysis of field notes, observations, meeting minutes, and key informant interviews revealed eight principles in SPIPA's CCC planning process (Figure), which we describe below along with quotes from tribal members interviewed and areas we identified where SPIPA could improve its CCC planning process.

**Figure.**
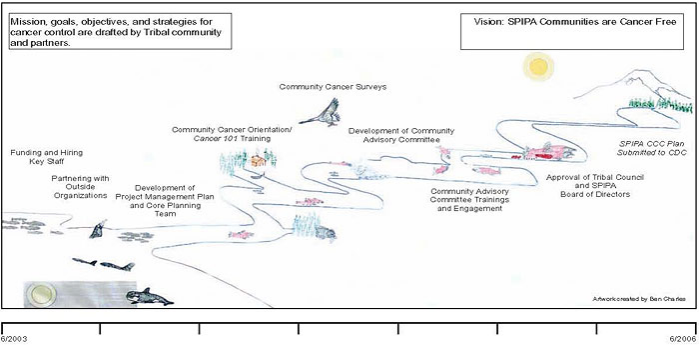
Mission, goals, objectives, and strategies for cancer control, South Puget Intertribal Planning Agency, comprehensive cancer control planning and community mobilization process, drafted by tribal community and partners.

**Table:** 

The figure is labeled “Mission, goals, objectives, and strategies for cancer control, South Puget Intertribal Planning Agency, comprehensive cancer control planning and community mobilization process, drafted by tribal community and partners." A figure note reads “SPIPA indicates South Puget Intertribal Planning Agency; CCC, Comprehensive Cancer Control; CDC, Centers for Disease Control and Prevention.”
The figure is a color landscape of the Washington coast and its wildlife with flow-chart text superimposed over the animals and features. The landscape depicts the migration of salmon from the sea, on the left-hand side of the drawing, upriver to spawning, on the drawing’s right side. A school of salmon in the sea is the first feature on the left, and over it is the caption, “funding and hiring key staff.” Swimming alongside the salmon is a group of dolphins and whales captioned, “partnering with outside organizations.” Next is another school of salmon approaching the river’s mouth from the sea. Its caption reads, “development of project management plan and core planning team.” A yellow cabin sits a short way up and alongside the river with the overhead caption, “community cancer orientation and Cancer 101 training.” Flying overhead is a large bird (a goose or a heron), captioned “community cancer surveys.” Pink salmon rounding a bend in the river are labeled with two captions, “development of community advisory committee” and “community advisory committee training and engagement.” The salmon arrive at their spawning site, and the caption overhead reads “approval of tribal council and SPIPA Board of Directors.” The river flows into a forested mountain on the far right of the drawing, which bears the caption, “SPIPA CCC submitted to CDC.”
Superimposed over the entire drawing are the words, “Vision: SPIPA communities are cancer free.”
Ben Charles created the original art for this figure.

### Funding and hiring key staff

Before seeking funding for its CCC program, SPIPA staff asked elders in the community about their needs for cancer education, screening, treatment, and support programs. These conversations led SPIPA staff to seek funding for cancer programs.

*Program  participant: My earliest conversations about cancer were with elders and others in the community. This was kind of the traditional start of a project in the SPIPA community. It starts with conversations about something the people have experienced and want. This led to the CDC grant process.*

SPIPA hired a CCC project coordinator to lead the CCC project shortly after funding was initiated. The project coordinator was an active member of a tribe served by SPIPA, was a respected tribal leader, and was experienced in program planning and community mobilization. Interviewees stated that experience, passion and enthusiasm for the work, and the ability to engage the communities were necessary staff attributes for the project to succeed.

*Program  participant: Well, I think it was a big project and I knew before we hired a project coordinator that this was an important project; despite the lack of expertise that I had, somehow I had to move it forward. As for myself, I think being able to feel comfortable with my project coordinator and not having to look over their shoulder, but just to provide guidance as needed and allow that person the freedom to move forward in getting the project off the ground* [*has been important*]. *That person has done a great job.*

### Partnering with outside organizations

Part of SPIPA's innovative planning process was to bring in three outside partners and their resources to assist a traditionally underfunded tribal system**.** SPIPA identified three key partners that shared its mission of reducing cancer disparities: the National Cancer Institute's Cancer Information Service–Northwest Region, Spirit of EAGLES, and the Alliance for Reducing Cancer, Northwest. The support these agencies provided was detailed in a memorandum of understanding at the start of this process.

As the CCC planning process gained momentum, the SPIPA Community Advisory Committee brought in other outside organizations, including the American Cancer Society, the American Lung Association, the Northwest Portland Area Indian Health Board, the Leukemia and Lymphoma Society, and the Washington Comprehensive Cancer Control Partnership. Although both the Washington State Department of Health and the Northwest Portland Area Indian Health Board have CCC funding, SPIPA had not been involved in their CCC planning efforts and wanted to develop an independent plan by and for SPIPA tribal members.

The interviewees expressed how important the partnerships have been and how vital it is for outside agencies to be respectful of each tribe's culture and strengths.

*Program  participant: I can't leave out the part either regarding the wonderful partnerships with other outside agencies and organizations who work with cancer. I think that we've done quite well in that arena to have them come and sit at the table with us and have them be respectful of our tribal communities and respectful of the fact those sitting on our community advisory committee may not have the education and background that they have working with cancer. But just the respectfulness says a lot and their willingness to provide training and to provide that training so that it's at a level of understanding for our community members.*

### Developing a project management plan and core planning team

SPIPA's CCC planning and community mobilization process marked the first time SPIPA used a systematic business model for planning a health or social services program. SPIPA's CCC coordinator used his expertise in business to apply the principles of core project management to the planning process. The project management plan consisted of identification of program goals and objectives, delegation of responsibilities and tasks, and a strategic plan to move the SPIPA communities in the direction of their stated vision, "cancer free tribal communities."

*Program  participant: When you assign these responsibilities, you have to select people who have some kind of business knowledge and/or desire to achieve those objectives.*

*You need to select those kinds of people based on what project you're doing and the areas they work in. It is really key to have a core planning team because one person can't do, or think of, all the things that need to be done.*

### Conducting community cancer orientations

Cancer community orientations were identified as a first step toward ensuring that the SPIPA CCC program truly was a community-driven process. The orientations were intended to increase community awareness of the CCC project, remove barriers and mitigate fears of cancer, increase participants' understanding of cancer by conducting a basic *Cancer 101* training, encourage tribal and community members to become members of the cancer advisory committee, and provide opportunities for community input on cancer control priorities. The significance of the orientations emerged in comments from interviewees regarding the positive effect of the sessions on generating interest among tribal communities in learning more about cancer in general and about SPIPA's CCC program. Participants expressed a need to repeat the cancer training and continue educational activities focusing on cancer prevention, screening, treatment, and survivor support.

*Program  participant: Since I'm the tribal clinic health director, I felt that it was important to attend. There was good energy in the community after the meeting and many discussions regarding cancer services at the tribal health clinic... a good opportunity to connect with the tribal community.*

An oncology nurse and a community health educator conducted education sessions, *Cancer 101*: *An Education and Training Program for American Indians and Alaska Natives* (7), at each of the community orientations. Designed by and for American Indians and Alaska Natives, *Cancer 101* is adaptable to the needs of the individual learner. The curriculum includes seven learning modules that cover cancer basics, such as how normal cells become cancerous, methods of early detection and screening, cancer treatment, and survivorship.

*Program participant: The education that I got through the* Cancer 101 *training really pinpointed some important issues that we needed to address with the committee, such as the types of cancers, the fact that there doesn't always have to be fear of cancer. Therefore, the information that was given to the community was helpful. It took a lot of the fear out of talking about cancer and for me personally. I was fearful to go get a PSA test because of all of the cancer in my family, and after the cancer training I didn't have that fear – I went and did it and found out that I was very healthy.*

### Conducting community cancer surveys

Community cancer surveys, distributed at the end of the community cancer orientations, provided the foundation for priorities in SPIPA's CCC plan (8). Over 400 surveys gave voice to the opinions and concerns of tribal and community members regarding cancer issues important to them. Overall priorities identified for inclusion in SPIPA's CCC plan were early detection of cancer and cancer screening, cancer education and prevention, cancer treatment, and cancer survivor support.

### Developing a community advisory committee

The SPIPA Community Advisory Committee provided the foundation for the CCC program and was its driving force. Committee membership consisted of representatives from each of the five tribes, SPIPA's CCC program staff, and staff from other partner organizations. Tribal representatives included tribal clinic staff, cancer survivors, tribal elders, and tribal leaders. With guidance from SPIPA CCC program staff, advisory committee members reviewed tribal cancer priorities, as stated in the community cancer surveys, and assisted in writing SPIPA's CCC plan. As a fundamental component of community-based participatory programs, all decisions were made in consultation with the community advisory committee — from broad decisions to specific ones (9). SPIPA's Community Advisory Committee enabled its CCC program staff to link with and build on the strengths and resources within the tribal communities. The community advisory committee met monthly.

*Program  participant: One strength is that this project has gotten a core group of tribal members to be involved to listen to their input. The core group that is involved usually doesn't attend community meetings, so it's good to see them involved. It may be due to them being affected by cancer.*

*This is the best organized committee that I've been involved with — it is really well organized. The agenda is sent before the meetings, and it's not written in stone. We're always free to talk about anything that we feel is important; we're led through the process, but not told how to do it. We are always allowed to have input . . . very little wasted time. We're always accomplishing something at the meetings, and you are never bored.*

### Training and engaging the community advisory committee

As the driving force behind the CCC program, the SPIPA Community Advisory Committee wanted to improve its leadership capacity with continued education. This education included advanced cancer training conducted by the same oncology nurse and community health educator who presented the *Cancer 101* sessions and training on goal and objective writing before the CCC plan was written. Interviewees who served as community advisory members indicated that the trainings improved their ability to assist family and community members with cancer questions and needs and helped prepare them to assist in writing SPIPA's CCC plan.

*Program  participant: The number one strength *[*of the CCC project*] *is that the SPIPA staff pulled in professional advisors to help us better understand cancer. Through the knowledge that we acquired through the educational sessions, it made it easy for us to put together a really good cancer plan.*

### Supporting the leadership of the communities involved

A final and critical component of SPIPA's CCC planning process was the support and approval of the SPIPA Board of Directors and each of the five tribal councils. Throughout the planning process, SPIPA's CCC coordinator updated tribal leaders through presentations to the board and tribal councils and through written updates. The SPIPA draft CCC plan was formally presented to the SPIPA Board of Directors and each of the five tribal councils for approval. The ongoing communication and updates with tribal councils and the board resulted in unanimous approval for the plan. In December 2005, the SPIPA Board of Directors approved the adoption and implementation of the SPIPA Comprehensive Cancer Control Plan.

*Program  participant: Every tribal leadership needs to be willing to make this a priority in order for this to succeed. Regular updates at tribal council meetings are important so that tribal council members can get the most appropriate people to be involved.*

### Areas of SPIPA's CCC planning process that could be improved

Many components of this project made it successful, although the interview data also identified two areas for improvement. Tribal health clinic provider input was sought from the beginning of planning, although few were actively involved throughout the process.

*Program  participant: I'd really like to see providers and health directors involved. More input from providers is needed.*

*To get more providers involved you need to be clear at the beginning that they are needed. If that would have been clear, then I would have had one of my providers involved.*

A SPIPA CCC medical advisory panel has now been formed as a venue to obtain provider input formally.

The second key area for improvement is increasing the involvement of men. Of the 27 community advisory committee members, 88% were women. A cancer workshop focusing on men is one of many activities that the SPIPA CCC Advisory Committee is currently working on to increase their involvement.

## Interpretation

Case study findings indicated that SPIPA will need to identify tribal cancer survivors and role models, including men, who can share their stories. Since the case study was completed, SPIPA has been successfully implementing its CCC plan and will continue to do so to reduce the burden of cancer among their tribal members.

The planning process is feasible, even for tribes with little experience and resources. The principles identified in the case study could help structure the cancer control planning process for other tribes.

## Figures and Tables

**Table.** Cultural Considerations in SPIPA's Comprehensive Cancer Control (CCC) Planning Process,^a^ Washington, 2005–2006

**Cultural Considerations**	**SPIPA's Response in CCC Planning**
Fear of the word "cancer" among tribe members; belief that cancer is a fatal disease and nothing can be done.	Mitigated by offering tribe-specific cancer training for tribal and community members. Tailored cancer education to meet the needs of the specific community.
History and perception that tribal communities lack ownership of health initiatives.	Acknowledged and honored tribes' desire and right to prioritize and address cancer issues and concerns. SPIPA emphasized cooperation and consensus-building with tribes to define CCC plan's goals, objectives, and strategies for achievement.
Essential need to have tribal community involved from the beginning in the CCC planning process.	Group decision-making process used rather than individual decision making.
Importance of ongoing communication with tribal councils; respect for tribal elders and tribal clinics.	SPIPA valued the wisdom that comes from age and shared experience.
Use of a combination of modern and traditional health care among more than 50% of SPIPA tribal members.	SPIPA valued traditional beliefs related to restoring health and wellness and incorporated these beliefs into the plan using a holistic approach to cancer.
Fear of the cost of cancer deterring tribe and community members from seeking cancer screening or follow-up services.	Careful attention placed on the unique challenges and disparities faced by community members in accessing adequate and timely care related to cancer.
Importance of religious and traditional beliefs to tribe members and of sharing meals together.	Traditional patterns of communication and celebration were acknowledged and incorporated into SPIPA's CCC planning process.

SPIPA indicates South Puget Intertribal Planning Agency.

a Source is SPIPA Community Cancer Survey, 2004, and SPIPA's CCC Case Study field notes and observations.
